# Medical intensive care unit clinician attitudes and perceived barriers towards early mobilization of critically ill patients: a cross-sectional survey study

**DOI:** 10.1186/1471-2253-14-84

**Published:** 2014-10-01

**Authors:** Sarah E Jolley, Janet Regan-Baggs, Robert P Dickson, Catherine L Hough

**Affiliations:** Division of Pulmonary and Critical Care Medicine, University of Washington, Seattle, WA USA; Department of Nursing, University of Washington, Seattle, WA USA; Division of Pulmonary and Critical Care Medicine, University of Michigan, Ann Arbor, MI USA; Division of Pulmonary and Critical Care Medicine, Harborview Medical Center, University of Washington, Campus Box 356522, Seattle, WA 98195-6522 USA

**Keywords:** Early mobilization, ICU acquired weakness, ICU mobility

## Abstract

**Background:**

Early mobilization (EM) of patients on mechanical ventilation (MV) is shown to improve outcomes after critical illness. Little is known regarding clinician knowledge of EM or multi-disciplinary barriers to use of EM in the intensive care unit (ICU). The goal of this study was to assess clinician knowledge regarding EM and identify barriers to its provision.

**Methods:**

Simultaneous cross-sectional surveys of medical ICU (MICU) nurses (RN)/physical therapists (PT) respondents and physician (MD) respondents in a single MICU at an academic hospital in Seattle, WA in 2010–2011. Responses were indicated on a 5 point Likert scale and reported as proportion of respondents agreeing or disagreeing. Chi-square testing and Fisher’s exact testing was performed to determine whether responses differed by duration of employment or prior EM experience.

**Results:**

A total of 120 clinicians responded to the survey (91 MDs (response rate 82% (91/111)), 17 RNs (response rate 22%, (17/78)), and 12 PTs (response rate 86%, (12/14)), overall response rate 86%). Most clinicians indicated knowledge regarding benefits of EM. More attending physicians reported knowledge of EM benefits, but also that risks of EM outweigh the benefits compared to trainees (p = 0.02 and 0.01). Clinicians across disciplines reported near universal agreement to use of EM for patients on MV, while the minority reported agreement to EM for patients on vasoactive agents. The most frequently reported cross-disciplinary barriers to EM were staffing and time. Risk of self-injury and excess work stress were indicated as barriers by RN and PT respondents.

**Conclusions:**

MICU clinicians, at our institution, reported knowledge of EM in the ICU. Staffing and clinician time were frequently identified cross-disciplinary barriers. Risk of self-injury and excess work stress were frequently reported RN and PT barriers.

**Electronic supplementary material:**

The online version of this article (doi:10.1186/1471-2253-14-84) contains supplementary material, which is available to authorized users.

## Background

Critically ill patients, particularly those requiring mechanical ventilation (MV), are prone to impairments in physical function associated with immobility and intensive care unit (ICU) acquired weakness. Functional impairments acquired during ICU hospitalization result in increased need for long term nursing care, greater risk of readmissions and reductions in health-related quality of life for ICU survivors [1–6]. There is an increasing body of literature reporting improvement in long-term function with provision of early physical and occupational therapy, so-called “early mobilization (EM)” initiated within 48 hours of mechanical ventilation and continued during the ICU stay [7–14].

These studies show that EM is safe, feasible, and results in significant improvements in delirium and function at time of hospital discharge [7–16]. Despite these potential benefits, provision of EM has not been widely adopted. A recent point prevalence study of 783 patients receiving mobility in ICUs across Germany reported that only 24% of the 185 patients receiving mechanical ventilation were mobilized out of bed on a single study date [17]. A similar single-day study in Australia and New Zealand reported that none of the 224 patients requiring mechanical ventilation were mobilized stood or ambulated over the course of the study day [18].

Quality improvement projects have attempted to understand whether clinician attitudes and education around EM serve as barriers to its delivery [13, 14, 19]. Results from these initiatives identify patient safety, staffing, and lack of clinician understanding as potentially important barriers to EM projects. We sought to understand whether these barriers to EM existed in our institution in order to provide a baseline understanding of our clinical practice around ICU mobility prior to implementation of a dedicated ICU mobility protocol.

Therefore, the aim of this study was to investigate whether clinicians in the medical intensive care unit at our institution are knowledgeable regarding the benefits of early mobilization and to identify perceived barriers to delivery of mobility in the ICU.

## Methods

We performed two simultaneous cross-sectional surveys of clinicians providing care in the medical intensive care unit (MICU) at our institution between July 2010 and July 2011. Our institution is a large, urban academic medical center in Seattle, WA. Physician subjects were identified during clinical rotations through the medical intensive unit. All rotating residents, fellows and attending physicians were invited to participate. Registered nurses and physical therapists working in the MICU were identified by the MICU nurse manager and the head of the physical therapy department. Participation was voluntary and each participant contributed one, distinct survey to the study. Physician participants were invited to participate at the start of clinical rotations through the MICU. Nursing and physical therapy participants were invited to participate during a regularly scheduled staff meeting. Potential participants were only contacted for potential participation on one occasion. Surveys were administered by an individual not involved in data analysis or primary study design with physician surveys administered on paper and nursing/physical therapist administered electronically. All surveys were anonymous and were collected in an anonymous manner.

Surveys were conducted prior to implementation of an ICU mobility program at our institution. At the time of survey administration, activity orders for critically ill patients required a physician orders with all activity performed by either the bedside nurse and/or a physical or occupational therapist who shared acute care floor and ICU duties. Physician surveys differed in wording from nursing/physical therapy surveys (Additional file 1).

Questionnaires were developed with physical and occupational therapy input and presented to focus groups of nurses, chief medical residents and pulmonary and critical care medicine physicians prior to survey administration. Clinical staff who participated in development of the survey did not participate as survey respondents. The questionnaires consisted of items assessing knowledge of potential benefits of early mobilization in the ICU, attitudes towards provision of therapy in the ICU and perceived barriers to delivery of EM [20]. Responses were indicated using a 5-point Likert scale: strongly agree, agree, neutral, disagree, and strongly disagree.

Early mobilization was defined as any activity beyond range of motion performed by a care provider (nursing, physical or occupational therapy) occurring within 48 hours of initiation of mechanical ventilation for our study purposes. Prior experience with early mobilization was defined as a respondent responding “yes” to the question, “Have you ever trained and/or worked at an institution that actively mobilizes patients receiving MV?” “Correct” answers for the knowledge questions were identified prior to survey administration. Reponses of strongly disagree or disagree were considered “correct” answers for the questionnaire item assessing whether range of motion was sufficient to maintain muscle strength. This was identified as a correct answer. Reponses of agree or strongly agree were considered “correct” answers to the questionnaire item assessing whether the use of early mobilization was associated with a decrease in duration of mechanical ventilation. This was chosen as a “correct” based on the results of the single randomized controlled trial using early mobilization as an intervention to date demonstrating a shorter duration of mechanical ventilation in patients receiving early ICU mobility [15]. For all other questions, a positive response was indicated by a response of “strongly agree” or “agree” with a negative response indicated by “neutral”, “disagree” or “strongly disagree”. A pre-populated list of potential barriers to ICU mobility was provided based on current known literature around ICU mobility for physician respondents which included the following: nursing time, respiratory therapy time, physical therapist availability, patient in procedures, over-sedation, mobility is not important in the ICU, delirium, access to specialized equipment, staff safety, patient safety, spine precautions, cost, therapy does not occur despite being ordered [13, 14, 19, 21–23]. Physician respondents could check all answers that applied and an optional write-in section was provided for barriers not covered by the populated list. A pre-populated list of potential staff barriers to ICU mobility was provided for nursing and physical therapist respondents which included the following: musculoskeletal injuries, fatigue, added work stress and the need to stay late in order to “catch up”. Nursing and physical therapist respondents could check all answers that applied.

Descriptive statistics were used to describe respondents. Likert scale responses were reported as frequencies and proportions. Chi-square testing was performed to test whether responses differed significantly among physician providers differing by level of training (faculty vs. trainee physicians) and differing by prior experience with early mobilization. Fisher’s exact testing was performed to test whether responses differed significantly among nursing and physical therapist providers differing by levels of experience (≥5 years vs. < 5 years) and differing by prior experience with early mobilization. A two-sided p-value of <0.05 was considered statistically significant for all analyses. Statistical calculations were performed using STATA 12.0 (Stata Statistical Software: Release 13. College Station, TX: StataCorp LP).

This study was conducted as part of an educational quality improvement initiative aimed at understanding institutional use of EM. The study was reviewed and considered non-Human Subjects research by the University of Washington Institutional Review Board. This study adheres to the Strengthening the Reporting of Observational Studies in Epidemiology (STROBE) guidelines (Additional file 2).

## Results

### Physician survey results

Ninety-one physicians completed a single survey (overall response rate of 82% (91/111)). Most physicians (79/91, 87%) were Internal Medicine or Pulmonary and Critical Care Medicine trainees (Table 1). Thirty-five percent (n = 31) of physicians surveyed reported past experience with EM in ICU patients. Most (n = 61, 68%) indicated that range of motion was insufficient to maintain muscle strength (n = 64, 70%) in critically ill patients and that EM reduces duration of MV (Table 2). Nineteen physicians surveyed (21%) agreed that the risks of EM in mechanically ventilated patients outweighs the potential benefits (Table 3). Faculty physicians were significantly more likely than trainees to indicate that EM reduces duration of mechanical ventilation (p = 0.02), but also more likely to report that the risks of EM outweigh the potential benefits of EM (p = 0.01). Indication of prior experience with EM was not associated with greater agreement regarding potential benefits (p = 0.12 and p = 0.24, respectively).

**Table 1:** **Characteristics of survey respondents and their prior experience with early mobilization, Seattle, WA, 2010-2011**

	N, (%)*
***Physician respondents:***	
Internal medicine trainee	66 (72)
Intern	34 (37)
Senior resident	32 (35)
Pulmonary and critical care trainee	11 (12)
Pulmonary and critical care attending faculty	12 (13)
No training level indicated	2 (2)
Reported prior experience with early mobilization	31 (35)
***Nurse respondents:***	
Medical ICU nurse ≥ 5 years of experience	12 (71)
Medical ICU nurse < 5 years of experience	5 (29)
Reported prior experience with early mobilization	5 (29)
***Physical therapist respondents:***	
Physical therapist ≥ 5 years of experience	10 (83)
Physical therapist < 5 years of experience	2 (17)
Reported prior experience with early mobilization	6 (50)

*Percentages may not add to 100% due to rounding.

**Table 2:** **Knowledge of potential benefits of early mobilization in the ICU by level of training**

Instrument item:	N, (% disagree)
Range of motion is sufficient to maintain muscle strength in the ICU	85 (71)
***Physician respondents:***	
IM intern (n = 34)	21 (63)
IM senior (n = 32)	20 (63)
PCCM fellow (n = 11)	10 (91)
PCCM attending (n = 12)	10 (83)
Level of training not identified (n = 2)	0 (0)
***MICU nurse respondents:***	
≥ 5 years of experience (n = 12)	9 (75)
< 5 years of experience (n = 5)	4 (80)
***Physical therapy respondents:***	
≥ 5 years of experience (n = 10)	9 (90)
< 5 years of experience (n = 2)	2 (100)
	**N, (% agree)**
Early mobilization reduces duration of mechanical ventilation	84 (70)
***Physician respondents:***	
IM intern (n = 34)	25 (74)
IM senior (n = 32)	19 (59)
PCCM fellow (n = 11)	7 (64)
PCCM attending (n = 12)	12 (100)
Level of training not indicated (n = 2)	1 (50)
***MICU nurse respondents:***	
≥ 5 years of experience (n = 12)	7 (58)
< 5 years of experience (n = 5)	3 (60)
***Physical therapy respondents:***	
≥ 5 years of experience (n = 10)	9 (90)
< 5 years of experience (n = 2)	1 (50)

**Table 3:** **Attitudes towards provision of early mobilization by level of training**

Instrument item:	N, (% agree)
The patient risk associated with mobilizing ventilated patients *outweighs* the benefits	22 (18)
***Physician respondents:***	
IM intern (n = 34)	6 (18)
IM senior (n = 32)	5 (16)
PCCM fellow (n = 11)	1 (9)
PCCM attending (n = 12)	6 (50)
Level of training not identified (n = 2)	1 (50)
***Nurse respondents:***	
≥ 5 years of experience (n = 12)	2 (17)
< 5 years of experience (n = 5)	1 (20)
***Physical therapy respondents:***	
≥ 5 years of experience (n = 10)	0 (0)
< 5 years of experience (n = 2)	0 (0)
I would agree to mobilization of a patient on vasopressor agents	34 (28)
***Physician respondents:***	
IM intern (n = 34)	7 (21)
IM senior (n = 32)	8 (25)
PCCM fellow (n = 10)	3 (30)
PCCM attending (n = 12)	2 (17)
Level of training not identified (n = 2)	0 (0)
***Nurse respondents:***	
≥ 5 years of experience (n = 12)	5 (42)
< 5 years of experience (n = 5)	2 (40)
***Physical therapy respondents:***	
≥ 5 years of experience (n = 10)	7 (70)
< 5 years of experience (n = 2)	0 (0)
I would agree to mobilization of a patient on mechanical ventilation	113 (94)
***Physician respondents:***	
IM intern (n = 34)	31 (91)
IM senior (n = 32)	32 (100)
PCCM fellow (n = 10)	9 (90)
PCCM attending (n = 12)	11 (92)
Level of training not identified (n = 1)	2 (100)
***Nurse respondents:***	
≥ 5 years of experience (n = 12)	12 (100)
< 5 years of experience (n = 5)	4 (80)
***Physical therapy respondents:***	
≥ 5 years of experience (n = 10)	10 (100)
< 5 years of experience (n = 2)	2 (100)

**Figure 1:**
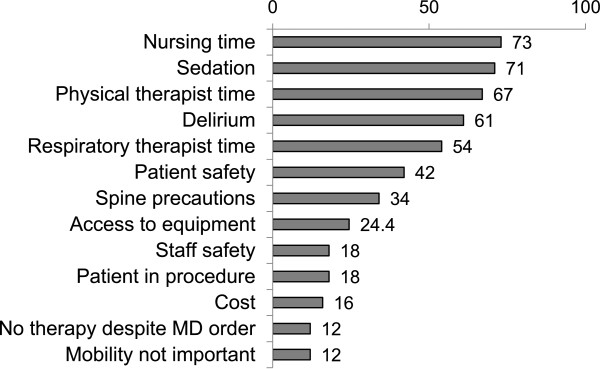
**Physician reported barriers to early mobilization of critically ill patients (n = 91, % reporting agree).**

Most physicians surveyed indicated that they would allow EM for patients on MV (n = 85, 94%) and most indicated they would be willing to alter their ventilator strategy (n = 64, 71%) to allow for EM. Forty-three percent (n = 39) of physicians indicated that they disagreed with EM for patients on vasopressor agents. Prior experience with EM was not associated with reported agreement with EM for patients on vasopressors (p = 0.53, experience vs. no experience). Eighty percent (n = 73) of physicians surveyed indicated that EM should occur automatically via nursing and PT protocols unless the physician specifically orders otherwise. Staffing, excessive sedation, delirium and patient safety were identified most frequently as barriers to EM (Figure 1). Lack of physician understanding of mobility and lack of data supporting mobility as an ICU therapy were rarely self-identified as barriers (n = 5, 6%).

### Nursing survey results

Seventeen registered nurses completed a single survey regarding EM (17/78, response rate 22%). Most nurse respondents (n = 12, 71%) reported greater than 5 years of employment in their current medical ICU. Most MICU nurses (n = 13, 76%) indicated that range of motion was insufficient to maintain muscle strength in critically ill patients and that use of EM reduces duration of MV (n = 10, 59%). Responses did not differ based on years of experience nursing (p = 0.67 and p = 0.69 respectively, ≥5 years vs. <5 years). Nurses who reported a history of prior experience with EM were more likely to report that reduction in duration of MV as a potential benefit (p = 0.04). Most MICU nurses surveyed (n = 16, 94%) agreed that it was possible to mobilize patients on MV while less than half (n = 7, 41%) agreed that it was possible to mobilize patients on vasopressor agents. Three nurses (18%) surveyed agreed that the risks of EM to patients outweighed the potential benefits (Table 3).

**Figure 2:**
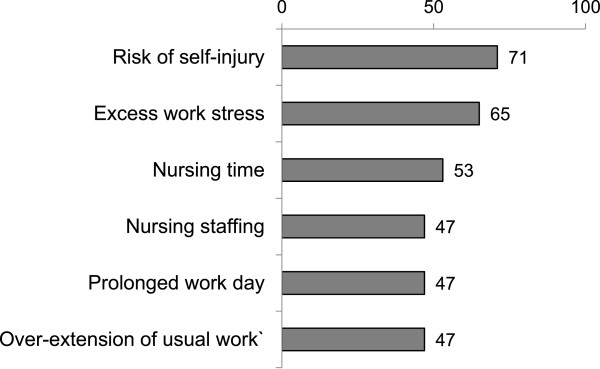
**Nursing reported barriers to early mobilization of critically ill patients (n = 17, % reporting agree).**

Risk of self-injury, excessive work stress and nursing time were the most frequently reported nursing barriers to EM (Figure 2). Most nurses (n = 15, 88%) indicated that the estimated time necessary for EM was between 16–45 minutes. Seventy-one percent (n = 12) of nurses surveyed indicated that use of EM placed staff at risk for musculoskeletal injuries and sixty-five percent (n = 11) indicated that EM added to overall work stress. Nearly half (n = 8, 47%) agreed that early mobilization contributed to prolonged work days and delay of usual work.

### Physical therapist survey results

Twelve physical therapists (PT) completed a single survey regarding EM (12/14, overall response rate 86%). Most PT respondents (n = 10, 83%) reported greater than 5 years of employment in their current medical ICU. Most physical therapists (n = 11, 92%) indicated that range of motion was insufficient to maintain muscle strength in critically ill patients and most (n = 10, 83%) indicated that EM reduces duration of MV. Responses to the knowledge questions did not differ by years of experience ((p = 0.83 and p = 0.32, respectively for ≥5 years vs. <5 years) as a physical therapist or by prior experience with EM (p = 0.50 and p = 0.23, respectively prior EM experience vs. no prior experience). All of the MICU PTs surveyed (n = 12, 100%) agreed that it was possible to mobilize patients on MV while fifty-eight percent (n = 7) agreed that it was possible to mobilize patients on vasopressor agents (Table 2).

**Figure 3:**
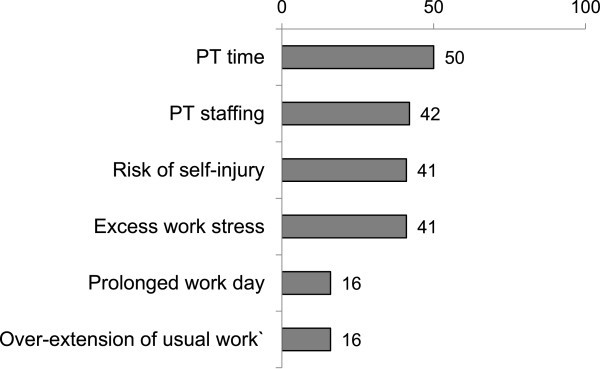
**Physical therapist (PT) reported barriers to early mobilization of critically ill patients (n = 12, % reporting agree).**

PT time, PT staffing and concern for staff-related injuries were the most frequently reported PT barriers to EM (Figure 3). Five PTs (41%) indicated that early mobilization added to overall work stress and two (16%) reported prolongation of their work day and delay of usual work resulting from EM.

## Discussion

Our study is the first to directly survey the full MICU care team involved in implementation of early mobilization (physicians, nurses and physical therapists) regarding knowledge and perceived barriers towards EM in critically ill patients. We targeted clinicians involved in patient care in the MICU given that the largest body of literature around EM to date exist for medical ICU patients, particularly patients diagnosed with respiratory failure [7, 11, 15]. We found that most clinicians (nurses, physical therapists and physicians) are knowledgeable regarding the potential benefits of EM including reductions in duration of mechanical ventilation and maintenance of muscle strength. Duration of employment and prior experience with EM were not associated with greater report of potential benefits across groups of MICU clinicians. Clinicians across all fields (physicians, nurses and PTs) reported acceptance of EM as a therapy in patients requiring MV, while most reported disagreement with use of EM in patients on vasoactive agents. Studies of patients receiving EM demonstrate that mobility is safe and feasible in patients on stable dose of vasoactive agents with very few adverse events [7, 11, 15, 24]. Further targeted education around appropriate patient selection and the role of mobility in patients with stable shock may enhance clinician acceptance of mobility in this subgroup of patients.

There were a number of shared barriers reported across all types of MICU clinicians including staffing and time. Finding the time and personnel necessary to mobilize can be a deterrent to implementation of mobility programs and an often reported concern in quality improvement studies targeting broader acceptance of mobility [12, 13, 19, 22, 25]. Our study findings suggest that clinicians think staffing is inadequate and nursing time insufficient. Centers have attempted to alter this perception by reprioritizing daily care routines to include mobility [7, 26, 27]. Simplified guidelines and electronic medical record triggers for therapy or mobility reduces nursing and therapy burden for initiating therapy shifting the focus towards a shared-multidisciplinary goal [19, 26]. Sharing patient and staff success stories across care disciplines and linking daily work to patient outcomes creates a sense of urgency and importance and potentially shifts clinician mindset from delivering a burdensome complex intervention to participating in a multidisciplinary team initiative [18]. Creation of dedicated mobility teams, dedicating PT resources solely to ICU patients via dedicated PTs or broader employment of therapy technicians and better defining the role of trainees in engaging in mobility may serve to increase delivery of EM.

Nursing and physical therapy staff highlighted a number of previously unreported barriers to EM that may be key to implementation of EM including risk of self-injury, perceived work stress and concerns over delay of usual work. Although studies demonstrate that EM is safe and feasible for patients, missed work related to staff musculoskeletal injury is under explored and may represent an important occupational health barrier to delivery of EM [28, 29]. Concerns over self-injury were greatest amongst nursing respondents with less than half of PTs (41%) reporting self-injury concerns and only 18% of physicians report staff safety as a barrier. Understanding care responsibilities towards EM may help to allay fears nursing staff have in mobilizing critically ill patients. Acceptance with mobility protocols and nursing confidence in ability to mobilize critically ill patients has been shown to directly correlate with the degree of ownership and responsibility nursing staff feel over mobility as an intervention [30]. Defining care roles and expectations between nursing and physical therapy staff may enhance overall access to EM.

Disconnect between physicians and nursing on patient-nurse safety risk may hinder efforts to optimize mobility if nursing staff perceive personal safety risks around mobilizing certain patient populations that are not conveyed to physicians. Targeted focus on mobility readiness that incorporate all members of the care team, including physicians, are needed in order to develop shared mental models with the patient and the mobilizing team [18]. Enhancing readiness by removing unnecessary devices, screening for staff safety risks in addition to patient safety readiness may reduce some adverse events while dedicated training in the use of mechanical training and mobility devices are shown to lessen staff physical burdens [29, 31]. Additionally, developing algorithms for how to incorporate mobility into usual care may serve to reduce some of the perceived work stress associated with mobility, while providing assistance in the form of nursing assistants or therapy/nursing teams may provide a sense of work distribution that lessens the perceived nursing burden. Further studies are needed that aim to better understand the burden of EM staff related adverse events, including staff injury and impact of mobility on clinician work stress.

Respondents in our study again identified sedation and delirium as barriers to EM. Prior mobility studies demonstrate a link between reducing levels of sedation in critically ill patients and subsequent increase in ICU mobility [13–15, 17–19]. Translating this into clinical practice broadly remains difficult. Recent studies have suggested that key ICU contextual factors including leadership, safety culture and knowledge deficits contribute to reduced implementation of coordinated awakening and mobility sessions [32, 33]. Incorporation of protocols linking daily interruptions of sedatives with mobility sessions may help to reduce over-sedation and delirium along with greater recognition of mobility as an important therapeutic option for management of ICU delirium.

Surprisingly, attending physicians were significantly more likely than trainees to report excess risk to early mobilization, potentially reflecting practice biases and reluctance to adopt new interventions too early. This sense of “clinical inertia” is reported in other care interventions and may serve as a safeguard given the uncertain nature of medicine [34, 35]. These result in the inability to advance care models and sub-optimal adherence to best practice guidelines [34, 35]. While clinical inertia is heavily studied in chronic disease management, it remains relatively unstudied in critical care. Our study suggests that “clinical inertia” may play an important role in implementation of new therapies in the intensive care unit.

Institution of checklists with targeted user feedback regarding performance adherence may aid in overcoming clinical inertia. Clinicians surveyed in our study appeared amenable to such interventions with 80% of physicians reporting willingness to relinquish decision making regarding early mobilization to multidisciplinary protocols. Further studies are needed to better define ideal care team models and care delivery systems for EM. Better definitions of care roles may allow for more targeted surveys of attitudes and behaviors that may influence implementation and adherence to mobility programs [36, 37]. Creation of multidisciplinary protocols may be necessary for broader implementation of ICU mobility given the reluctance of physicians regarding this intervention. This type of model has demonstrated success in increasing mobility access rates in a number of focused single-center quality improvement programs [13, 14, 19, 22, 27, 38]. Education regarding appropriateness, safety and promotion of early mobilization of critically ill patients may enhance physician and nurse acceptance.

This study has a number of important limitations. First, the surveyed sample represents a small sample of ICU care providers from a single institution. Our results are subject to selection bias regarding clinicians who opted to participate in the survey, particularly around the nursing respondents given the low response rate. While focus groups and pilot testing were utilized for survey development, our surveys were limited in length and scope by timing of administration and feasibility. Potential interactions may exist between survey questions that may introduce response bias. Additionally, answers to “knowledge” questions may be influenced by the limited early mobilization literature and potential reduced generalizability from EM clinical trials. Our results represent a baseline for our institution, but cannot fully address all potential attitudes and barriers towards early mobilization. Larger survey-based studies are needed to understand how pervasive these attitudes are across the broader ICU mobility. Finally, our survey did not survey administrative leaders. Understanding administrator attitudes towards EM is necessary when addressing hospital-level barriers including resource allocation and staffing. The strength of our study is that it is the first to broadly survey all MICU care clinicians within an institution to better understand cross-disciplinary care concerns around EM. As our study was focused at a single institution, it is possible that the results reflect local culture limiting generalizability and the low response rate by nursing providers limits generalizability of the nursing results. However, selection of an average MICU in a large academic hospital mirrors the care settings represented in most EM literature to date.

## Conclusions

Medical intensive care unit clinicians at our institution, overall, were knowledgeable regarding the potential benefits of early mobilization for critically ill patients. Clinicians across MICU care disciplines (physicians, therapists, nurses) agreed to EM of patients on MV, but disagreed regarding EM in patients on vasoactive agents. Attending physicians were more likely to report the risks of EM outweighed the benefits compared to trainees, despite being more likely to identify potential benefits to EM. Staffing and clinician time were the most frequently reported barriers to EM across disciplines. Risks of self-injury, excess work stress and delay of usual care were identified as nursing and PT specific barriers to EM. Additional research on appropriate patient selection for EM and risks of EM to staff combined with enhanced education, may be necessary for broader implementation of EM programs across medical intensive care units.

### Key messages

-  Medical intensive care unit nurses, physical therapists and physicians are knowledgeable regarding potential benefits to early mobilization including reductions in duration of mechanical ventilation and maintenance of muscle strength.
-  Attending physicians were significantly more likely to report knowledge of benefits to early mobilization and to report that the risks of mobilization outweigh the potential benefits compared to trainees.
-  Physical therapy and nursing staffing and time were the most frequent physician reported barriers to early mobilization.
-  Excess work stress and risk of self-injury were commonly reported nursing and physical therapy reported barriers to early mobilization.

## Electronic supplementary material

Additional file 1: **Clinician attitudes survey supplement 1.** (DOCX 15 KB)

Additional file 2: **STROBE Statement—Checklist of items that should be included in reports of** ***cross-sectional studies.*** (DOC 79 KB)

## Abbreviations

EM: Early mobilization
PT: Physical therapy
ICU: Intensive care unit
MICU: Medical intensive care unit
MV: Mechanical ventilation
RN: Nurse.
