# Supplementary material for: Medical intensive care unit clinician attitudes and perceived barriers towards early mobilization of critically ill patients: a cross-sectional survey study
Source: BMC Anesthesiol. 2014 Oct 1;14:84. doi: 10.1186/1471-2253-14-84 (PMC4192294; doi:10.1186/1471-2253-14-84)
Supplement: Supplementary file 1 — Additional file 1: Clinician attitudes survey supplement 1. (DOCX 15 KB) [file 12871_2014_298_MOESM1_ESM.docx]

| **Demographic Information:** |
| --- |
| 1. Please indicate your specialty and level of training. 2. Have you ever trained and/or worked at an institution that actively mobilizes patients receiving mechanical ventilation? |
| **Knowledge of early mobilization:** |
| 1. Range of motion is sufficient to maintain muscle strength in the ICU. 2. Early mobilization reduces duration of mechanical ventilation. |
| **Attitudes towards early mobilization:** |
| 1. The patient risk associated with mobilizing ventilated patients outweighs the benefits. 2. Mobilization of ICU patients should occur automatically via a nursing and PT protocol unless the physician specifically orders otherwise. 3. I would agree to mobilization of a patient on mechanical ventilation. 4. I would agree to mobilization of a patient on vasopressors. |
| **Behaviors regarding early mobilization:** |
| 1. I would be willing to decrease sedation to facilitate mobilization of my ICU patients. 2. I would be willing to alter the mechanical ventilation settings of my ICU patients to facilitate mobilization. 3. Please identify what you think are significant barriers to mobilizing medical ICU patients (*check all that apply*): nursing time, respiratory therapy time, physical therapist availability, patient in procedures, over-sedation, mobility is not important in the ICU, delirium, access to specialized equipment, staff safety, patient safety, spine precautions, cost, therapy does not occur despite being ordered, other (specify) |
| **Table S1: Physician questionnaire questions** |
| **Demographic information:** |
| 1. What is your profession? 2. How many years have you been in your chosen profession? 3. How many years have you worked in a critical care environment? 4. Have you ever ambulated (walked) a patient who was receiving mechanical ventilation? |
| **Knowledge of early mobilization:** |
| 1. Range of motion exercise (active or passive) is sufficient to maintain muscle strength in critically ill patients. 2. It is possible to mobilize a patient receiving mechanical ventilation on a stable dose of IV vasoactive medication. 3. Potential effects of early mobilization for patients receiving mechanical ventilation includes faster ventilator weaning. |
| **Attitudes towards early mobilization:** |
| 1. The patient risk associated with mobilizing ventilated patients outweighs the benefits. 2. Staffing is adequate to mobilize patients receiving mechanical ventilation in the ICU. 3. I have enough time to help mobilize a patient receiving mechanical ventilation once per day. 4. The risks to staff of mobilizing mechanically ventilated ICU patients outweighs the benefits to the patients. |
| **Behaviors regarding early mobilization:** |
| 1. The risks **to staff** when mobilizing patients receiving mechanical ventilation includes: musculoskeletal injury, fatigue, added work stress, need to stay late in order to “catch up” |

**Table S2: Nursing and Physical Therapist questionnaire questions**
